# A Comparison of the Efficacy of High-Dose Vitamin C Infusion and Thiamine (Vitamin B1) Infusion in Patients With Sepsis: A Prospective Randomized Controlled Trial

**DOI:** 10.7759/cureus.75296

**Published:** 2024-12-07

**Authors:** Prashant K Mishra, Atit Kumar, Sonali Agrawal, Deepika Doneria, Raghvendra Singh

**Affiliations:** 1 Anaesthesiology and Critical Care, Uttar Pradesh University of Medical Sciences, Etawah, IND

**Keywords:** crp level, sepsis, sofa score, thiamine, vitamin c

## Abstract

Background and objective

Vitamin C and thiamine possess properties that may mitigate the harmful effects of sepsis. However, there is a dearth of studies in the literature comparing these two vitamins with each other and with a placebo regarding their efficacy against sepsis. This study aimed to investigate the outcomes associated with high-dose infusions of vitamin C and thiamine in septic patients, thereby seeking to contribute valuable insights into the optimal management of sepsis. The primary objective was to compare the sequential organ failure assessment (SOFA) score and C-reactive protein (CRP) level improvement on Day Six among the vitamin C, thiamine, and placebo groups after the intervention for five days.

Methodology

This prospective randomized comparative study involved 75 patients. Patients were randomized into three groups of 25 each. The first group received high-dose vitamin C infusion along with standard treatment for sepsis; the second group received high-dose thiamine infusion along with standard treatment for sepsis; and the third group, the placebo group, received only standard treatment for sepsis for five days. The SOFA score, CRP level, and other parameters were evaluated on Day Six.

Results

The SOFA score (p=0.043) and CRP level (p=0.0161) on Day Six were lower in the vitamin C group than in the placebo group. The CRP level on Day Six was significantly lower in the thiamine group than in the placebo group (p=0.016). The duration of vasopressor therapy was significantly lower in the vitamin C group than in the placebo group (p=0.0276) and the thiamine group (p=0.0236).

Conclusions

Based on our findings, vitamin C infusion helps improve the SOFA score and CRP level in sepsis patients. It can also decrease the duration of vasopressor therapy and serious adverse events whereas thiamine can reduce CRP levels in these patients.

## Introduction

Sepsis is a significant cause of mortality in hospitals worldwide, accounting for one-third to half of all deaths [1]. Globally, sepsis is estimated to affect 15-19 million individuals annually, with higher incidence rates in developing countries [2]. Infectious agents that provoke an immune response can lead to systemic inflammatory response syndrome and potentially progress to septic shock [3]. Sepsis is defined as a life-threatening organ dysfunction resulting from a dysregulated host response to infection. Clinically, organ dysfunction is characterized by an increase in the sequential organ failure assessment (SOFA) score [4]. Lactate is the most commonly utilized biomarker for identifying sepsis. However, additional biomarkers could augment lactate's effectiveness. These include markers indicative of sepsis' hyper-inflammatory phase, such as pro-inflammatory cytokines and chemokines, and proteins like C-reactive protein (CRP) and procalcitonin [5].

Patients in the ICU who are on a ventilator for more than two days are at increased risk of developing ventilator-associated pneumonia (VAP) [6], which is the second most common hospital-acquired infection and can also precipitate sepsis. Sepsis can result in acute lung injury and acute respiratory distress syndrome (ARDS) caused by tissue inflammation and the production of reactive oxygen species, leading to free radical damage [7]. Vitamin C is a powerful antioxidant that effectively neutralizes the various free radicals produced in the body. It is essential for synthesizing collagen, vasopressin, cortisol, and catecholamines, which are crucial for the survival of patients with sepsis [8]. It may reduce lung injury associated with sepsis by inhibiting the accumulation of cytokines and neutrophils.

Due to the substantial depletion of vitamin C during severe inflammatory responses, sepsis patients may require considerably higher doses [9]. Thiamine is a cofactor for pyruvate dehydrogenase enzyme, essential for converting pyruvate to the acetyl-coenzyme A required in the Krebs cycle. A lack of thiamine prevents pyruvate from entering the tri-carboxylic acid cycle, which causes anaerobic metabolism [10]. Thiamine deficiency occurs in about one-third of septic patients. The supplementation of ascorbic acid and thiamine in sepsis patients continues to be a compelling therapeutic method due to its potential advantages, affordability, and positive safety profile.

The rationale behind comparing the efficacy of high-dose vitamin C infusion and thiamine infusion in sepsis lies in the evaluation of the potential benefits of these vitamins in mitigating the harmful effects of sepsis. Vitamin C has been studied for its antioxidant properties and its role in modulating inflammation, potentially reducing the severity of sepsis and improving patient outcomes. Similarly, thiamine is essential for cellular metabolism and has been hypothesized to have protective effects in sepsis by supporting cellular energy production and reducing oxidative stress.

## Materials and methods

Study design and setting

This prospective randomized comparative study was conducted from April 2024 to September 2024 after obtaining approval from the institutional ethical committee of Uttar Pradesh University of Medical Sciences, Saifai (approval no: 44/2022-23. This study is registered in the Clinical Trials Registry-India (CTRI): CTRI/2024/03/063852.

Patient selection and randomization

**Figure 1:**
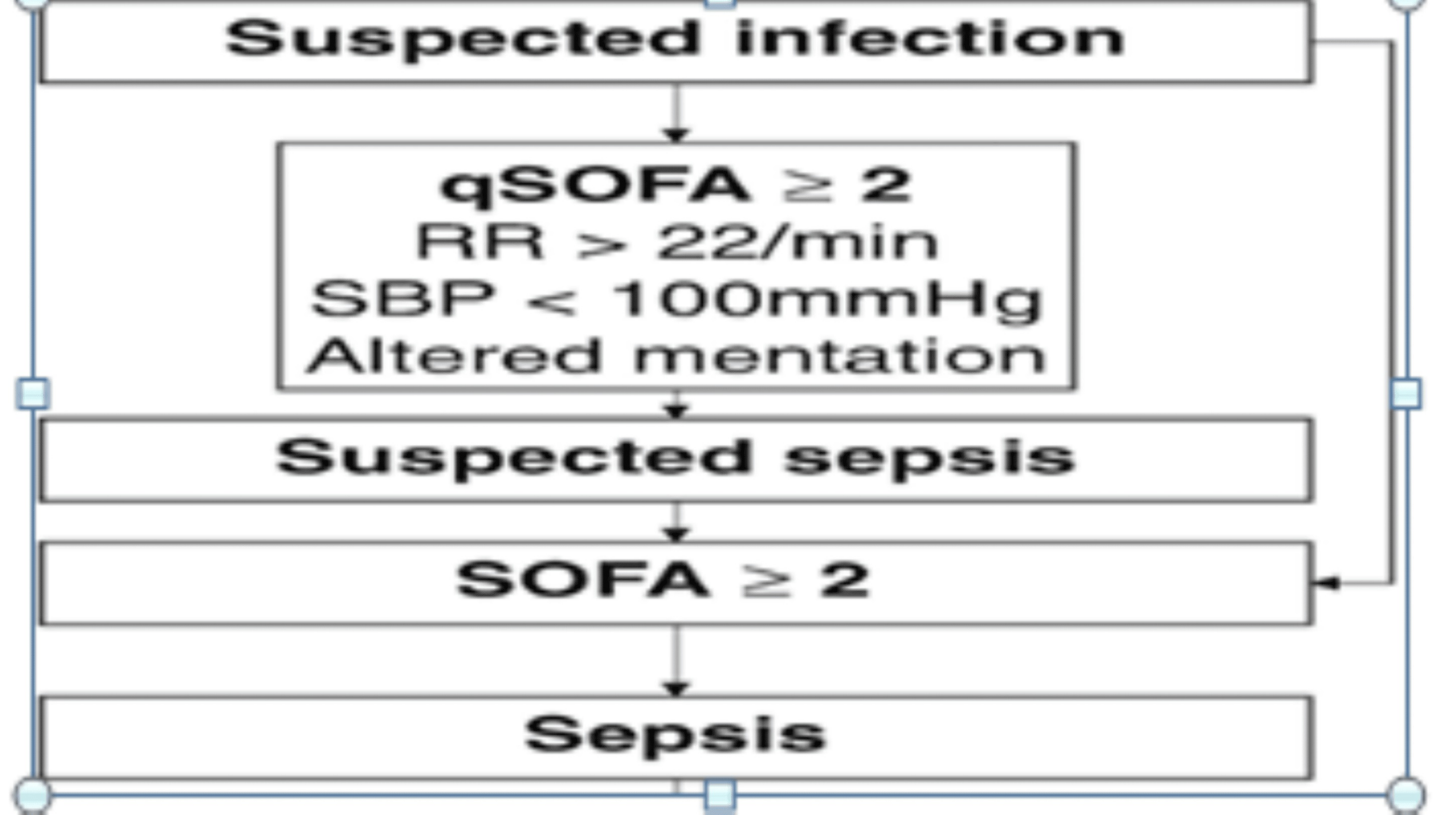
Sepsis criteria qSOFA: quick Sequential Organ Failure Assessment; RR: respiratory rate; SBP: systolic blood pressure

**Figure 2:**
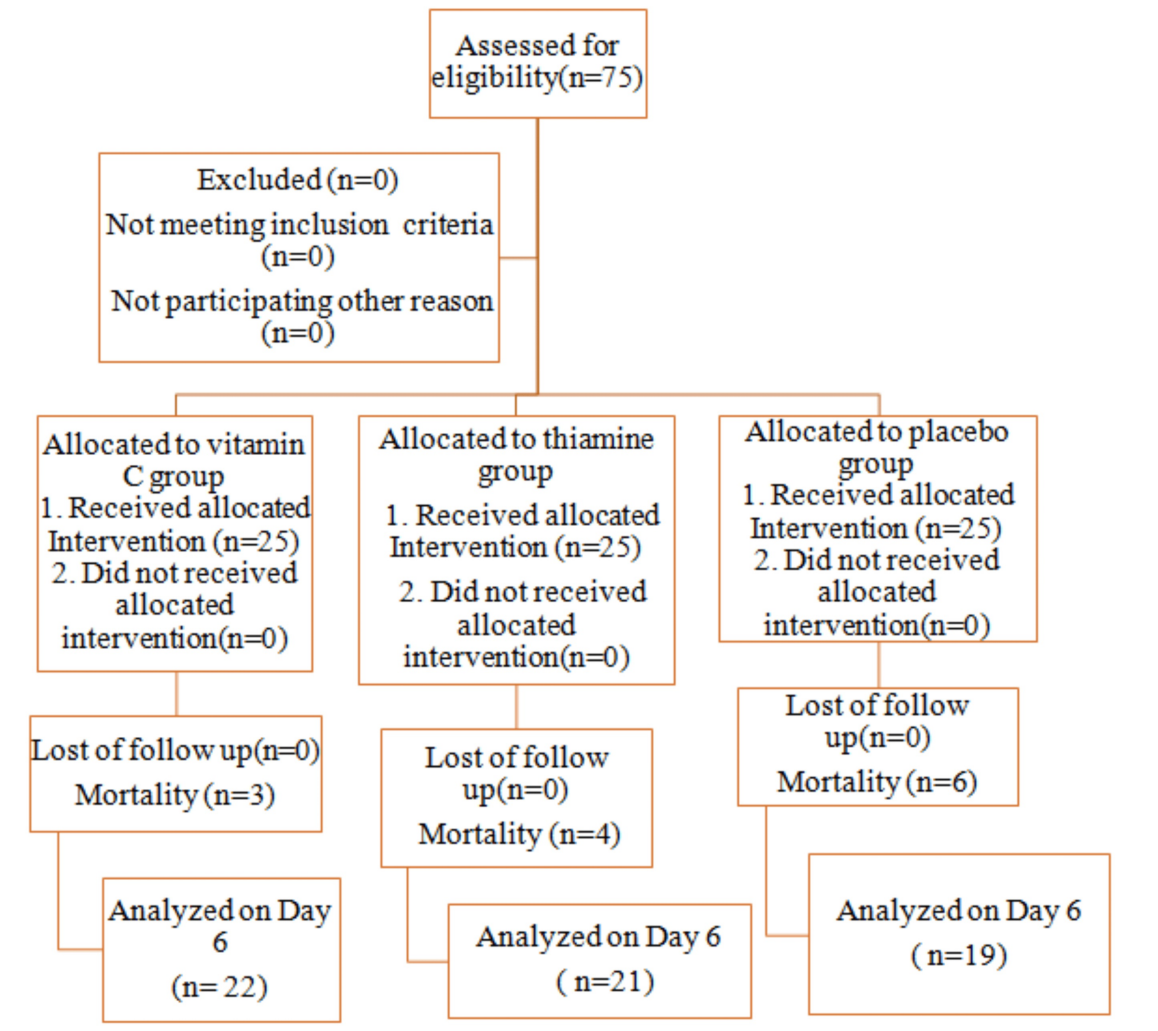
CONSORT flow diagram depicting patient selection and classification CONSORT: Consolidated Standards of Reporting Trials

Patients with suspected infection, having temperature >38℃ (fever), heart rate >100 beats per minute (tachycardia), and quick SOFA (qSOFA) score ≥2 were included in the study (Figure 1). The patients included were surgical patients (postoperative cases of perforation peritonitis and intestinal obstruction). They were on similar board-spectrum antibiotic therapy. Patients had SOFA scores>2 but <8 and a similar level of CRP on Day One. Randomization was performed using an online computer-generated random number table and group allocation was done through a sequentially numbered sealed opaque envelope technique. A total of 75 patients were assigned to three groups of 25 patients each (Figure 2). The patients and the observer who recorded all data were unaware of the therapy used in any of the groups; data were collected by a junior resident posted in the department of anesthesia of UPUMS, Saifai.

Based on a study by Marik et al. [11], the sample size was calculated with an alpha error of 0.05, a confidence interval of 95%, and a power of 80%. The minimum sample size was determined to be 24.4, and hence we included 25 patients in each group. Patients between the ages of 18 and 65 with a BMI between 18-30 kg/m² were included. Pregnant or breastfeeding females, patients with cancer as the cause of sepsis, those with kidney disease and alcoholism, and immune-compromised patients were excluded from the study.

Objectives

The primary objective was to compare the SOFA score and CRP level on Day One and Day Six. The secondary objective was to compare incidences of VAP if the patient required mechanical ventilation, the duration of vasopressor therapy if it started after inclusion in the study, mortality during the study, or any serious adverse event like ARDS.

Study groups

Group A received intravenous supplementation of vitamin C at a dose of 2.5 grams every eight hours (total dose of 7.5 grams per day) for five days. This was administered as an infusion over 30 minutes, mixed in a 50 ml solution of either 5% dextrose in water or normal saline. This was in addition to the standard treatment for sepsis. Group B received intravenous supplementation of 250 mg thiamine every eight hours (total dose of 750 mg per day) for five days. This was administered as an infusion over 30 minutes, mixed in a 50 ml solution of either 5% dextrose in water or normal saline. This was in addition to the standard treatment for sepsis. Group C received only a 50 ml solution of either 5% dextrose in water or normal saline in addition to the standard treatment for sepsis, with no additional supplementation.

Data collection

Blood samples were drawn on Day One and Day Six from all three groups for routine investigations to determine the SOFA score and CRP levels. The Day Six SOFA scores of all the patients as well as the differences between Day One and Day Six SOFA scores were calculated. The outcomes were recorded in terms of improvement in SOFA scores, CRP levels, vasopressor therapy, and duration of ventilator-free days if the patient required mechanical ventilation. Other parameters like the incidence of VAP, length of hospital stay, and serious adverse events like ARDS were also recorded.

Statistical analysis

The statistical analysis was done using SPSS Statistics version 20.0 (IBM Corp., Armonk, NY). The quantitative variables were expressed as mean ± standard deviation (SD) and compared with the unpaired t-test. The Mann-Whitney Test was used to compare non-nominal variables and Chi-square/Fisher’s exact test for qualitative variables. A p-value <0.05 was considered statistically significant, and the data were stored on an MS Excel spreadsheet.

## Results

Table 1 shows the mean SOFA scores on Day One and Day Six among Groups A, B, and C. There was a statistically significant difference in the SOFA scores on Day Six in Group C compared with Group A. However, there was no statistically significant difference in the SOFA scores on Day One among the groups in the study.

**Table 1:** Comparison of mean SOFA scores on Day One and Day Six among the groups ^*^Statistically significant Group A: patients receiving vitamin C. Group B: patients receiving thiamine. Group C: patients receiving placebo SD: standard deviation; SOFA: sequential organ failure assessment

Group	A (n=25), mean ± SD	B (n=25), mean ± SD	C (n=25), mean ± SD	P-value		
				A vs. B	B vs. C	C vs. A
SOFA score on Day One	6.52 ± 1.12	6.48 ± 1.36	6.64 ± 1.19	0.9101	0.660	0.7151
SOFA score on Day Six	3.91 ± 2.76	4.71 ± 3.13	6.00 ± 3.64	0.3786	0.2357	0.0434^*^

Table 2 shows the mean CRP levels on Day One and Day Six among Groups A, B, and C. There was a statistically significant difference in the CRP level on Day Six in Group C vs. A and B vs. C. However, there was no statistically significant difference in the CRP level on Day One among the groups, showing the comparability of groups in the study.

**Table 2:** Comparison of CRP level (mg/dl) on Day One and Day Six among the groups *Statistically significant Group A: patients receiving vitamin C. Group B: patients receiving thiamine. Group C: patients receiving placebo CRP: C-reactive protein; SD: standard deviation

Group	A (n=25), mean ± SD	B (n=25), mean ± SD	C (n=25), mean ± SD	P-value		
				A vs. B	B vs. C	C vs. A
CRP level on Day One, mg/dl	188.84 ± 73.03	190.40 ± 48.63	184.04 ± 77.63	0.9295	0.7300	0.8228
CRP level on Day Six, mg/dl	128.41 ± 112.11	138.33 ± 57.58	228.89 ± 141.08	0.7190	0.0023^*^	0.0161^* ^

Table 3 shows the duration of vasopressor therapy among Groups A, B, and C. There was a statistically significant difference in the duration of vasopressor therapy in Group C vs. A and B vs. A. The Standard deviation value was greater than the mean in Group A because most of the patients had vasopressor therapy for zero days and some patients had vasopressor therapy for five to six days.

**Table 3:** Comparison of duration of vasopressor therapy (in days) among the groups ^*^Statistically significant Group A: patients receiving vitamin C. Group B: patients receiving thiamine. Group C: patients receiving placebo SD: standard deviation

Group	A (n=25), mean ± SD	B (n=25), mean ± SD	C (n=25), mean ± SD	P-value		
				A vs. B	B vs. C	C vs. A
Duration of vasopressor therapy, days	1.64 ± 2.00	2.88 ± 1.74	2.90 ± 1.80	0.0236^*^	1.00	0.0276^*^

Table 4 shows the incidence of VAP, mortality, and serious adverse effects such as ARDS events in Groups A, B, and C. ARDS was significantly less in Group A. There was no statistically significant difference in the incidence of VAP and mortality among the groups.

**Table 4:** Distribution of the incidence of VAP, mortality, and ARDS in patients in groups A, B, and C *Statistically significant Group A: patients receiving vitamin C. Group B: patients receiving thiamine. Group C: patients receiving placebo ARDS: acute respiratory distress syndrome; VAP: ventilator-associated pneumonia

Group	Group A (n=25)		Group B (n=25)		Group C (n=25)		P-value
	N	%	N	%	N	%	
Incidence of VAP	8	32.00	9	36.00	12	48.00	0.481
Mortality	3	12.00	4	16.00	6	24.00	0.521
ARDS	7	28.00	12	48.00	17	68.00	0.018^*^

## Discussion

Demographic characteristics like age, sex, and BMI were comparable among all three groups in this study. On Day Six, the mean SOFA score was 3.91 (SD: 2.76) in the vitamin C group, 4.71 (SD: 3.13) in the thiamine group and 6.00 (SD: 3.64) in the placebo group. In intergroup comparisons, there was a statistically significant difference between the vitamin C group and the placebo group (p<0.05), while no statistically significant difference was seen between the vitamin C group and the thiamine group, and the thiamine group and the placebo group (p>0.05). A study by Luo et al. [12] showed improvement in SOFA scores with vitamin C treatment in a statistically significant manner, which is consistent with our study.

The mean CRP level on Day Six in the vitamin C group was 128.41 mg/dl (SD:112.41), while it was 138.33 mg/dl (SD=57.5) in the thiamine group, and 228.89 mg/dl (SD: 141.0) in the placebo group. In the intergroup comparison, there was a statistically significant difference between the thiamine group and the placebo group as well as the vitamin C group and the placebo group (p<0.05). However, no statistically significant difference was seen between the vitamin C group and the thiamine group (p>0.05). El Driny et al. [13] investigated the effects of high-dose vitamin C infusion in septic patients requiring mechanical ventilation in a double-blind randomized controlled trial and demonstrated significant improvement in CRP level, which aligns with our findings.

The mean duration of vasopressor therapy in the vitamin C group was 1.64 days (SD: 2.00), while it was 2.88 days (SD: 1.74) in the thiamine group and 2.90 days (SD: 1.80) in the placebo group. In another intergroup comparison, there was a statistically significant difference between the vitamin C and the thiamine group comparison as well as the vitamin C group and the placebo group (p<0.05). However, there was no statistically significant difference between the thiamine group and the placebo group (p>0.05). Liang et al. [14] conducted a systematic review and meta-analysis to assess the effect of intravenous vitamin C on adult septic patients and found that intravenous vitamin C significantly decreased the duration of vasopressor use, which is in line with our results.

The mean duration of ventilator-free days was 2.48 days (SD: 2.31) in the vitamin C group, while it was 2.64 days (SD: 2.33) in the thiamine group and 2.31 days (SD: 1.76) in the placebo group. In intergroup comparison, no statistically significant difference was found (p>0.05). This is consistent with the above-mentioned study by Liang et al. [14]. VAP was seen in eight patients in the vitamin C group, nine patients in the thiamine group, and 12 patients in the placebo group. A comparison among all the groups showed a p-value >0.05, which was statistically insignificant. A study by El Driny et al. [13] investigated the effects of high-dose vitamin C infusion in septic patients requiring mechanical ventilation. It found that early administration of high-dose vitamin C by intravenous infusion, in conjunction with standard sepsis treatment, led to a significant reduction in the incidence of VAP.

ARDS was seen in seven patients in the Vitamin C group, 12 patients in the thiamine group, and 17 patients in the placebo group. The intergroup comparison showed a p-value <0.05, demonstrating statistical significance. A study by Fowler [15] on vitamin C use in sepsis-induced ARDS found that vitamin C can serve as a potent anti-inflammatory agent and alter the proinflammatory states that lead to ARDS; a treatment regimen of intravenous vitamin C may help attenuate the lung injury of ARDS. Mortality was seen in three patients in the vitamin C group, four in the thiamine group, and six in the placebo group. Intergroup comparison showed a p-value >0.05, showing a lack of statistical significance. Zeng et al. [16] conducted a meta-analysis focusing on intravenous high-dose vitamin C monotherapy for sepsis and septic shock patients, which revealed a significant decrease in short-term, all-cause mortality in patients with sepsis receiving high-dose vitamin C. El Driny et al. [13] investigated the effects of high-dose vitamin C infusion in septic patients requiring mechanical ventilation in a double-blind randomized controlled trial and demonstrated significant improvements in 28-day mortality. In our study, patients were followed up for only up to six days, which may be the reason why the mortality result was insignificant.

Limitations

This study has a few limitations. Our sample size of 25 participants per group may have been too small to detect minor differences among interventions, potentially limiting the generalizability of the results. Moreover, the study duration might not have been sufficient to observe long-term outcomes and effects of vitamin C and thiamine infusion beyond the immediate treatment period.

## Conclusions

In our study, the SOFA score, CRP level, duration of vasopressor therapy, and serious adverse events were significantly lower in the vitamin C group than in the thiamine group and placebo group. The duration of vasopressor therapy and CRP levels were significantly lower in the thiamine group than in the placebo group. High-dose vitamin C infusion was more effective than high-dose thiamine infusion in sepsis as it reduced the SOFA score, CRP level, duration of vasopressor therapy as well as serious adverse events. Thiamine infusion reduced CRP level and duration of vasopressor therapy with no effect on SOFA score and serious adverse events. Overall, these findings suggest that vitamin C infusion is better than thiamine infusion in septic patients when used along with standard treatment for sepsis.
